# Redox-Linked Domain Movements in the Catalytic Cycle of Cytochrome P450 Reductase

**DOI:** 10.1016/j.str.2013.06.022

**Published:** 2013-09-03

**Authors:** Wei-Cheng Huang, Jacqueline Ellis, Peter C.E. Moody, Emma L. Raven, Gordon C.K. Roberts

**Affiliations:** 1 Henry Wellcome Laboratories for Structural Biology, Department of Biochemistry, University of Leicester, Henry Wellcome Building, Leicester LE1 9HN, UK; 2 Department of Chemistry, University of Leicester, Leicester LE1 7RH, UK

## Abstract

NADPH-cytochrome P450 reductase is a key component of the P450 mono-oxygenase drug-metabolizing system. There is evidence for a conformational equilibrium involving large-scale domain motions in this enzyme. We now show, using small-angle X-ray scattering (SAXS) and small-angle neutron scattering, that delivery of two electrons to cytochrome P450 reductase leads to a shift in this equilibrium from a compact form, similar to the crystal structure, toward an extended form, while coenzyme binding favors the compact form. We present a model for the extended form of the enzyme based on nuclear magnetic resonance and SAXS data. Using the effects of changes in solution conditions and of site-directed mutagenesis, we demonstrate that the conversion to the extended form leads to an enhanced ability to transfer electrons to cytochrome *c*. This structural evidence shows that domain motion is linked closely to the individual steps of the catalytic cycle of cytochrome P450 reductase, and we propose a mechanism for this.

## Highlights

• Cytochrome P450 reductase is in equilibrium between compact and extended forms
• The position of the equilibrium depends on the redox state of the enzyme
• The extended form favors electron transfer to cytochrome *c*
• Domain motion is linked closely to the individual steps of the catalytic cycle

## Graphical Abstract

**Figure:**
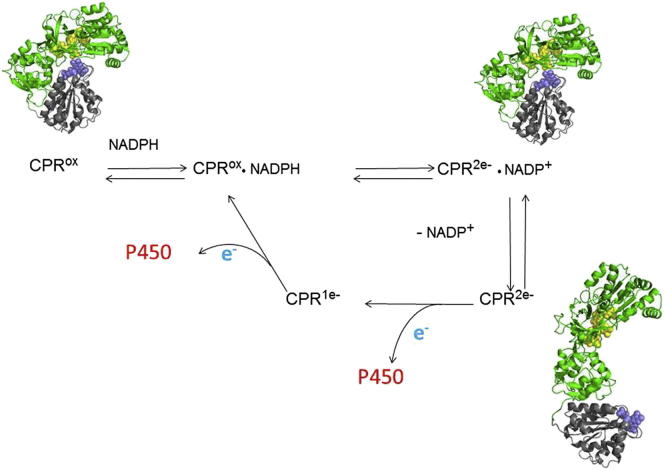

## Teaser

Solution scattering experiments by Huang et al. connect domain movement in cytochrome P450 reductase to individual steps in the catalytic cycle. The domain movement necessary for enzyme turnover is not achieved simply by random diffusion; rather, it is linked closely to the individual steps of the catalytic cycle.

## Introduction

Proteins show internal motions over a wide range of timescales (picoseconds to seconds) and amplitudes (0.01 to >50 Å), and it is clear that at least some of these motions contribute to biological function—notably, in enzyme catalysis. The idea of an energy landscape for a folded protein is well established (Frauenfelder et al., 1991), and recent structural evidence has shown that some of the conformational substates of unliganded enzymes correspond to states along the reaction coordinate, leading to the idea of the importance of “conformational selection” (e.g., Boehr et al., 2009; Hammes et al., 2011; Ma and Nussinov, 2010). Many of the motions involved in these processes are relatively local—notably, loop movements—and for a few enzymes, the “choreography” of these motions in the catalytic cycle has been studied in detail (Boehr et al., 2010; Eisenmesser et al., 2005; Henzler-Wildman et al., 2007). However, there are instances where much larger scale movements of whole domains are essential in enzyme turnover (e.g., Qi and Hayward, 2009; Reger et al., 2008; Wolthers et al., 2010) and, notably, in electron transfer pathways. Electron transfer is generally carried out by proteins associated in large dynamic complexes; in such systems, domain motion can be required to provide access for the protein partner(s) to the redox center(s), and the importance of large-scale protein dynamics in biological electron transfer is now recognized (e.g., Danyal et al., 2010; Lennon et al., 2000; Toogood et al., 2007).

There is evidence for domain motion in the family of diflavin reductases, which includes cytochrome P450 reductase (CPR) (Iyanagi et al., 2012; Wang et al., 1997; Xia et al., 2011b), nitric oxide synthase (Haque et al., 2012; Iyanagi et al., 2012; Stuehr et al., 2009), and methionine synthase reductase (Wolthers et al., 2007). These enzymes have three domains: an FMN-binding domain, related to flavodoxins; an FAD- and NADPH-binding domain, related to ferredoxin/flavodoxin reductases; and a “linker” domain, the FMN domain being connected to the linker and FAD domains through a highly flexible “hinge.” CPR is a key component of the P450 mono-oxygenase system of the endoplasmic reticulum, which plays a central role in drug metabolism (Riddick et al., 2013). Cytochromes P450 (P450s) catalyze the insertion of one atom of molecular oxygen into their substrates with the reduction of the other atom to water, a reaction requiring two electrons which, in the case of the drug-metabolizing P450s, are supplied by CPR (Iyanagi et al., 2012; Murataliev et al., 2004). CPR accepts electrons from the obligatory two-electron donor NADPH on to its FAD cofactor and transfers them via its FMN cofactor to a wide range of different P450s. The two electrons are donated one at a time at two distinct steps in the P450 reaction cycle (Makris et al., 2005; Murataliev et al., 2004).

The conformation of truncated soluble CPR seen in the crystal (Wang et al., 1997; Xia et al., 2011b) is well suited for electron transfer from FAD to FMN, as the two isoalloxazine rings are <4 Å apart. However, in this conformation, it is difficult to see how the large P450 (or cytochrome *c*, widely used as a surrogate redox partner for studies in solution) could approach close enough to the FMN for electron transfer to occur (Aigrain et al., 2012). The implication is that the domains of CPR must move relative to one another to allow access of the redox partners to the FMN, and there is circumstantial evidence for this from our own and other laboratories (Aigrain et al., 2012; Ellis et al., 2009; Grunau et al., 2007; Gutierrez et al., 2002, 2003; Hamdane et al., 2009; Hay et al., 2010; Iyanagi et al., 2012; Jenner et al., 2011; Pudney et al., 2011; Xia et al., 2011a). We have shown (Ellis et al., 2009; Jenner et al., 2011) that, in solution, the wild-type human enzyme can adopt an extended conformation resulting from a substantial domain movement. We now report detailed studies of the conformational equilibrium of CPR in solution using small-angle X-ray scattering (SAXS) and small-angle neutron scattering (SANS) methods and present a model of the extended state based on nuclear magnetic resonance (NMR) and SAXS data. The effects of redox state and coenzyme binding on the position of the equilibrium allow us to relate the domain movement to individual steps in the catalytic cycle of the enzyme.

## Results

### Reduction Converts CPR from a Compact to an Extended Conformation

**Figure 1:**
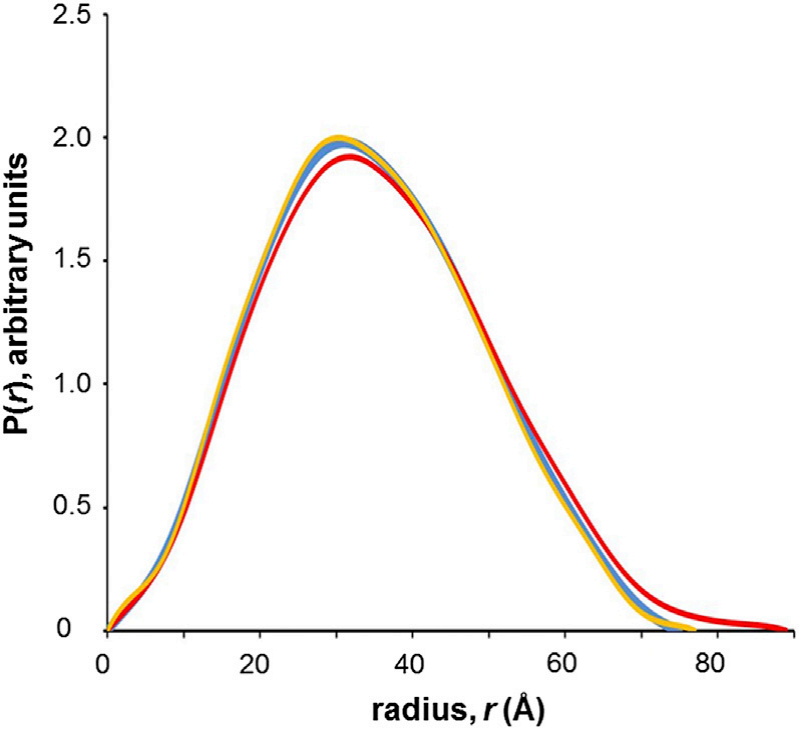
Intraparticle Distance Distribution Functions Derived from SAXS of CPR in the Oxidized and Reduced States Blue, oxidized enzyme; red, enzyme reduced to the two-electron level by careful anaerobic titration with dithionite, monitored by optical absorption spectroscopy; yellow, enzyme reduced to the two-electron level by addition of stoichiometric NADPH. See also Figures S1 and S2.

We have shown, using SAXS and ion-mobility mass spectrometry, that CPR having both flavins in the oxidized state (CPR^ox^) exists in equilibrium between compact and extended conformations (Ellis et al., 2009; Jenner et al., 2011). These SAXS experiments, carried out at the Science and Technology Facilities Council (STFC) Synchrotron Radiation Source (Daresbury, UK) using a wavelength of 1.53 Å and long exposure times (25–60 min), suggested that CPR^ox^ consisted of a roughly 50:50 mixture of compact and extended conformations (Ellis et al., 2009), whereas the mass spectrometry experiments suggested a much smaller (∼15%) proportion of the extended conformation (Jenner et al., 2011). Figure 1 shows the intraparticle distance distribution function, P(*r*), for CPR^ox^ and for two-electron-reduced CPR (CPR^2e−^, produced by addition of either dithionite or NADPH), calculated from SAXS experiments carried out using a wavelength of 0.931 Å and a much shorter exposure time (0.5 s); very similar results were obtained using a continuously flowing sample to further minimize exposure. (The scattering curves are shown in Figure S1 available online.) The data for CPR^ox^ are consistent with a compact conformation, whose radius of gyration (*R*_*g*_) of 26.4 Å (Table 1) is closely similar to the value of 26.6 Å for the *R*_*g*_ of the hydrated molecule calculated from the crystal structure (Wang et al., 1997). The results of these SAXS experiments are clearly more similar to those of our mass spectrometry experiments (Jenner et al., 2011) on CPR^ox^ than to our earlier SAXS results (Ellis et al., 2009). There is evidence that flavins in protein crystals can be reduced by photoelectrons produced by exposure to high X-ray doses (Berkholz et al., 2008; Johansson et al., 2010; Kort et al., 2004), and this has been demonstrated by Raman spectroscopy (Røhr et al., 2010). We have now shown by optical spectroscopy that the flavins of CPR are also reduced during X-ray exposure in solution (Figure S2). This suggests that the SAXS results reported earlier (Ellis et al., 2009) for “oxidized” CPR may, in fact, have corresponded to a mixture of oxidized enzyme and enzyme reduced to the disemiquinone level, because of partial reduction by X-ray-induced photoelectrons, leading to a substantial overestimate of the proportion of the extended conformation in the oxidized state.

To avoid any possibility of partial reduction during the experiment, we carried out SANS experiments on CPR; the *R*_*g*_ values for CPR^ox^ and CPR^2e−^ are given in Table 1, and the Guinier plots are shown in Figure S3. The SANS observations confirm that, in the oxidized state, CPR adopts a compact conformation. the direct comparison of the R_g_ values obtained by SAXS and SANS is complicated by the different effects of hydration in the two experiments (Perkins, 2001; Svergun et al., 1998).

Comparison of the P(*r*) curves in Figure 1 clearly shows a longer “tail” to the distribution at large *r* values for CPR^2e−^ produced by dithionite reduction, leading to a value for the maximum dimension, *D*_*max*_, of 80 Å, as compared to 73 Å for the oxidized enzyme (Table 1). The SANS data confirm that CPR^2e−^ adopts an extended conformation, with an *R*_*g*_ as much as 4 Å greater than in the oxidized state (Table 1).

Both SANS experiments and short-exposure SAXS experiments demonstrate that reduction of CPR to the two-electron-reduced state leads to a significant increase in the proportion of an extended conformation, showing clearly that the domain movement in this enzyme is linked to the catalytic cycle.

### Effect of Coenzyme Binding on the Position of the Conformational Equilibrium

It is notable that reduction of CPR to the two-electron level by stoichiometric NADPH rather than by dithionite yields a more compact conformation of the enzyme (Figure 1; Table 1). Under the conditions of these experiments, the product NADP^+^ is expected to remain bound to the enzyme (the dissociation constant, K_D_, of NADP^+^ for CPR^ox^ is 53 nM; Grunau et al., 2006), giving CPR^2e−^ ⋅ NADP^+^. Indeed, addition of NADP^+^ to CPR^2e−^ (obtained by dithionite reduction) leads to a more compact state of the enzyme (Table 1). By contrast, reduction by stoichiometric NADH yields an *R*_*g*_ value similar to that obtained by dithionite reduction (Table 1); NAD^+^ binds only weakly, so that reduction by NADH or by dithionite would both yield coenzyme-free CPR^2e−^.

These experiments indicate that, while two-electron-reduction of CPR in the absence of coenzyme shifts the conformational equilibrium toward an extended conformation, coenzyme binding shifts it back toward a compact conformation.

### A Model for the Structure of the Extended Conformation of CPR

We have obtained an approximate model of the extended conformation of oxidized CPR using the program HADDOCK (Dominguez et al., 2003), allowing the FMN domain to move relative to the rest of the protein, subject to the constraints of (1) the length of the flexible “hinge” between the domains, (2) the molecular envelope obtained from SAXS experiments at high ionic strength (discussed later), and (3) a number of identified residues whose environment differs between intact CPR and the isolated FMN domain, as indicated by differences in NMR chemical shifts (Figure S4).

**Figure 2:**
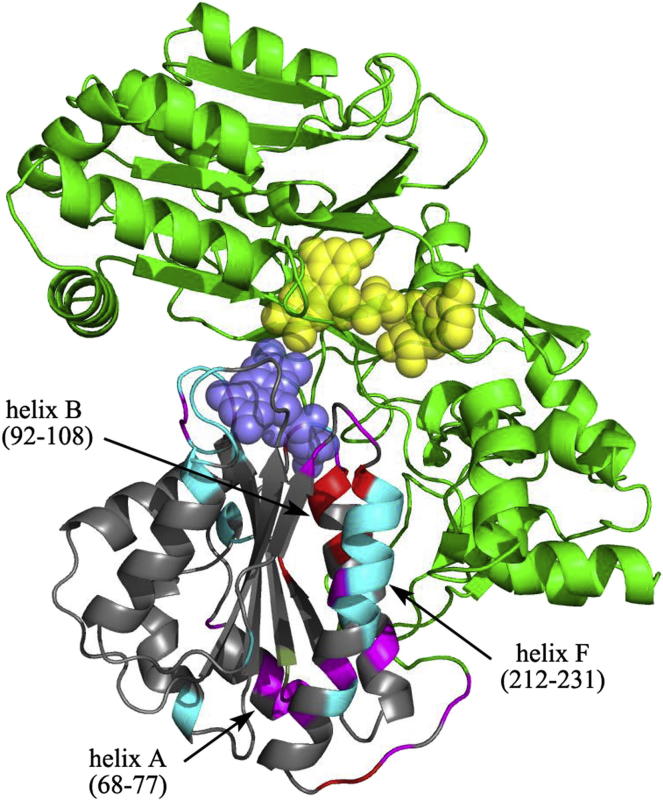
Chemical Shift Differences for Residues in the FMN Domain between the Intact Enzyme and Isolated Domain, Mapped onto the Crystal Structure The linker and FAD domains are shown in green, and the flavin cofactors in space-filling representation. In the FMN domain, residues showing combined (^1^H and ^15^N) backbone chemical shift differences between the isolated domain (Barsukov et al., 1997; Ellis et al., 2009) and the intact enzyme (see Experimental Procedures) of >0.3 ppm, >0.15 ppm, and <0.15 ppm are indicated in red, magenta, and gray, respectively. Residues in the FMN domain whose cross-peaks are too broad to detect are indicated in cyan. See also Figure S4.

The structure of the isolated FMN domain (Zhao et al., 1999) is closely similar to that of the domain in CPR (root-mean-square deviation = 0.25 Å), so we hypothesized that these shift differences, shown on the crystal structure in Figure 2, report on domain movements not on structural changes within the domain. Residues in loops close to the FMN, notably 85–92 and 139–144, whose environment in CPR will clearly be different from that in the isolated domain, show significant shift differences. However, the largest chemical shift differences, almost 1 ppm, together with extensive line broadening, are seen for residues in the first half of helix 212–231 (helix F), which are largely solvent exposed in the crystal structures of both CPR (Xia et al., 2011b) and the FMN domain (Zhao et al., 1999). More modest shift differences are also seen for residues in helix A, at the far end of the domain from the FMN. These observations suggest the existence in solution of some population of a conformation differing from that seen in the crystal structure.

**Figure 3:**
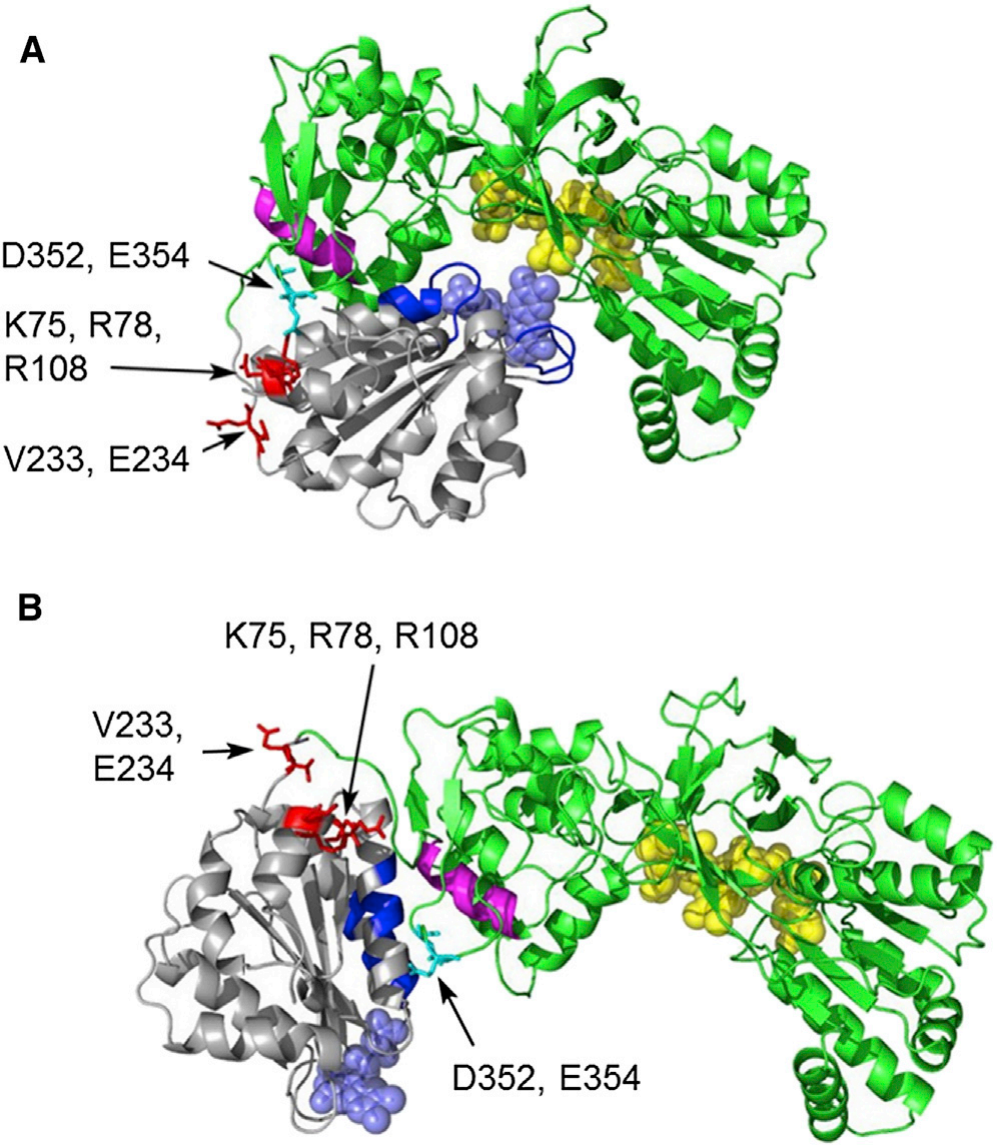
The Compact and Extended Conformations of CPR (A) Compact conformation, represented by the crystal structure. (B) Model of the extended conformation of CPR, derived as described in the text. The flavin cofactors are shown in space-filling representation (FMN, slate blue; FAD, yellow). The linker and FAD domains are shown in green, except for helix I in the linker domain, discussed in the text, which is shown in magenta. The FMN domain is in gray, except for residues involved in interdomain contacts in the two conformations, which are shown in dark blue. Mutated residues are shown, with their side chains, in red; residues D352 and E354, discussed in the text, are shown in cyan. See also Figure S5.

The model of the extended conformation is compared to the crystal structure in Figure 3. Regions of the FMN domain involved in interdomain interactions are shown in dark blue. In the crystal structure, these primarily involve loops around the FMN, but, in addition, two residues near the N termini of helices B and F (E96, E216) interact with residues (R285 and T386) at the N terminus of helix I in the linker domain (Figure 3, shown in magenta). In the model of the extended conformation, these three helices interact much more extensively, involving almost the whole length of helix I. The residues of these helices, particularly those involved in these interdomain interactions, are highly conserved (Figure S5). Thus, the loss of the interdomain interactions of the loops around the FMN is compensated by the much more extensive interactions between helices B and F in the FMN domain and helix I in the linker domain. Comparing our model of the extended conformation to the crystal structure, it is apparent that a major difference between the two arises from a rotation of the FMN domain over the surface of the linker domain, leading to the FMN becoming solvent accessible (Figure 3).

### The Extended Conformation of CPR Is Functionally Important

**Figure 4:**
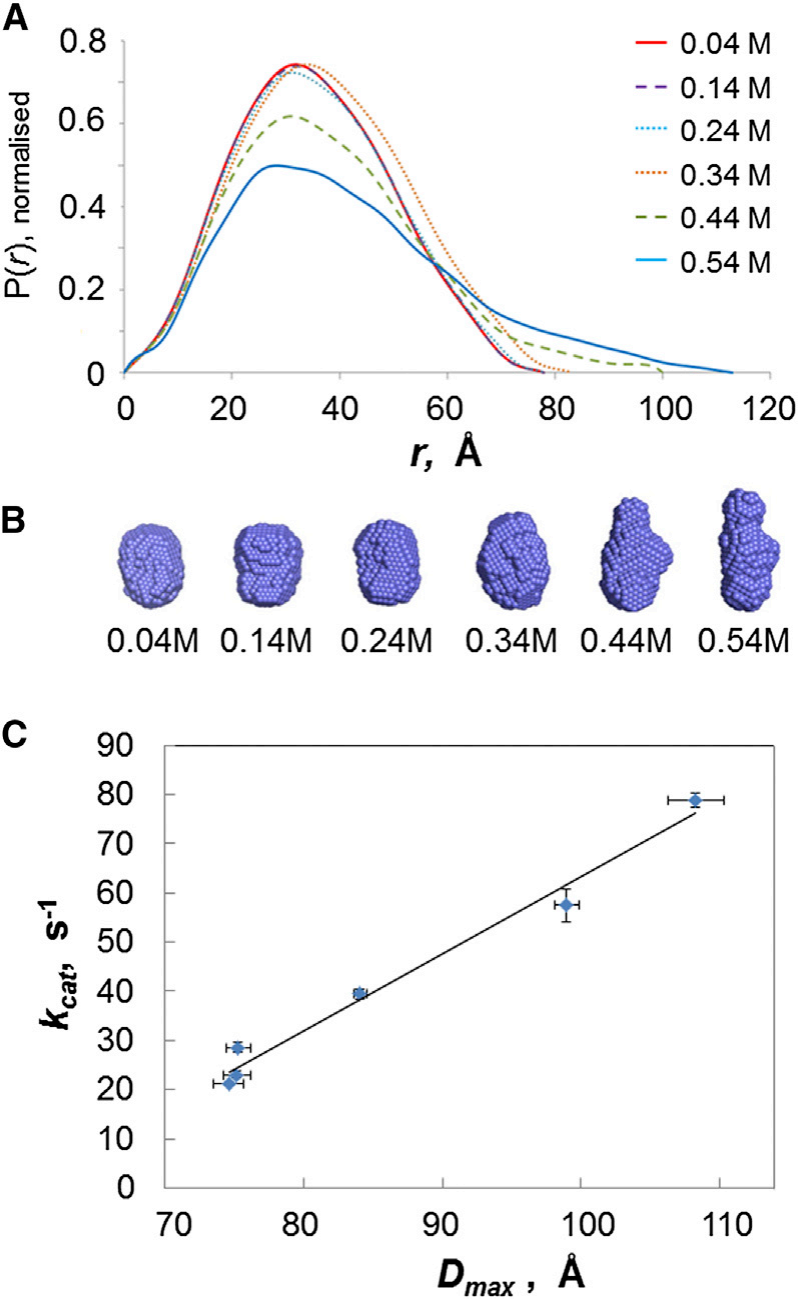
Effects of Ionic Strength on the Shape and Catalytic Activity of CPR (A) Intraparticle distance distribution functions of CPR at different ionic strengths (100 mM BES, pH 7.0, plus varying concentrations of NaCl). (B) Molecular shapes derived from the solution scattering data using the program GASBOR. (C) Correlation between the *D*_*max*_ values and the k_cat_ values for cytochrome *c* reduction at different ionic strengths (*R*^*2*^ = 0.98). See also Figure S6 and Table S1.

Increasing ionic strength affects the rates of CPR-catalyzed reduction of P450s or of cytochrome *c* (e.g., Davydov et al., 2000; Jang et al., 2010; Sem and Kasper, 1995), leading to an increase both in catalytic rate constant, k_cat_, and in Michaelis-Menten constant, K_M_, for cytochrome *c* (Figure S6). There is evidence that ion-pair interactions are involved in CPR-cytochrome interactions (Bridges et al., 1998; Shen and Kasper, 1995; Zhao et al., 1999), so that an increase in K_M_ with increasing ionic strength would be expected, although the reason for the increase in k_cat_ is less apparent. The crystal structures of CPR (Wang et al., 1997; Xia et al., 2011b) show that the domain-domain interface around the FAD and FMN cofactors includes a number of ion-pair interactions, raising the possibility that increasing ionic strength would affect the position of the conformational equilibrium. SAXS experiments show that CPR becomes more elongated at high ionic strength. Increasing the ionic strength up to 0.24 M has no significant effect, but further increases lead to a marked elongation of the molecular envelope until, by *I* = 0.54 M, *R*_*g*_ has increased from 26.4 Å to 32.5 Å and *D*_*max*_ has increased from 74 Å to 108 Å (Figure 4A; Table S1). The effect of high salt is completely reversible on decreasing the salt concentration by dialysis. These results are consistent with the idea that oxidized CPR exists in equilibrium between compact and extended conformations, the position of which is sensitive to the ionic strength of the solution. As the ionic strength is varied, we observe a striking correlation between k_cat_ and *D*_*max*_ (Figure 4C); this can readily be understood if the extended form of the enzyme, favored at high ionic strength, is required for intermolecular electron transfer.

To further test the importance of the extended conformation for the activity of CPR, we have constructed mutants involving residues at interdomain interfaces or in the flexible hinge. The positions of the residues involved are shown in Figure 3, and the shapes of these mutants and their kinetic constants for cytochrome *c* reduction are given in Table 2. The mutation V233P/E234P, involving two residues at the very beginning of the flexible “hinge,” remote from the linker and FAD domains, leads to a substantial increase in the *R*_*g*_ and *D*_*max*_ values, indicating a larger population of the extended conformation, a 3-fold decrease in the K_M_ for cytochrome *c* and a slight increase in k_cat_. The FMN domain of CPR has clusters of negatively charged residues around the FMN binding site and a striking patch of positively charged residues at the opposite end of the domain. In the crystal structure, two of these latter residues, K75 and R78, make salt bridges to two residues, E354 and D352, respectively, on the surface of the linker domain, while in our model of the extended conformation, these two salt bridges are broken and the relevant residues are >15 Å distant from one another (Figure 3). We have shown earlier that neutralization of the latter residues in the mutant D352N/E354Q leads to a substantial increase in the population of the extended conformation, from 15% to 65% (Jenner et al., 2011). Charge reversal mutations in the interacting positively charged residues in the FMN domain, in the mutant K75E/R78E/R108Q, lead to a substantial increase in *R*_*g*_, consistent with an increased population of the extended conformation (Table 2). It is striking that this mutant also shows a 5-fold decrease in the K_M_ for cytochrome *c* and a doubling of k_cat_ for cytochrome *c* reduction, providing further support for the importance of the conformational equilibrium for the activity of the enzyme.

Both the effects of increasing ionic strength and those of mutation show a correlation between an increase in the extended conformation and increased activity in cytochrome *c* reduction, demonstrating that this conformation plays a key role in the catalytic cycle.

## Discussion

The data presented here show clearly that, in solution, CPR exists in equilibrium between a compact conformation and a more extended conformation and that this equilibrium is linked to redox state and catalytic turnover.

### The Conformational Equilibrium in CPR^ox^

For the oxidized enzyme, the *R*_*g*_ values and the molecular envelope derived from our solution scattering experiments are essentially consistent with the compact conformation seen in the crystal. In a recent NMR study of the oxidized enzyme, Vincent et al. (2012) showed that a partial set of ^1^H-^15^N residual dipolar couplings was consistent with the compact conformation as seen in the crystal, with a maximum of 5%–7.5% of an extended conformation. Our earlier ion-mobility mass spectrometry studies (Jenner et al., 2011) indicated that, in CPR^ox^, the extended conformation represents approximately 15% of the population, in broad agreement with these results. The difference from our conclusions from earlier SAXS experiments (Ellis et al., 2009) arises from partial reduction of the flavins by X-ray-induced photoelectrons in those experiments. Comparison with our SANS data shows that this can be avoided by the use of short wavelengths and, particularly, short exposure times in SAXS.

There have been two reports of crystal structures of “open” conformations of CPR: a rat CPR mutant, ΔTGEE, in which four residues were deleted from the hinge region (Hamdane et al., 2009), and a chimeric enzyme consisting of the FMN domain from yeast CPR with the hinge and the linker and FAD domains from human CPR (Aigrain et al., 2009). In both these structures, residues that show chemical shift differences between the isolated FMN domain and intact CPR remain solvent exposed, and the calculated *R*_*g*_ value for the chimeric enzyme is much greater than any value measured in the present work. Thus, our model of the extended conformation derived from data on wild-type CPR is not identical to either crystal structure. It is, however, consistent with the behavior of the mutants K75E/R78E/R108Q and D352N/E354Q, in which the interdomain salt bridges K75-E354 and R78-D352 seen in the compact conformation are abolished, since in our model of the extended conformation these pairs of residues are distant from one another (Figure 3).

Increasing the ionic strength leads to a marked increase in the maximal dimension of the oxidized enzyme, consistent with a shift in the position of the equilibrium between compact and extended conformations as the salt concentration is increased, and this is also shown by mass spectrometry (Jenner et al., 2011). All our data, from SAXS, SANS, mutagenesis, and, particularly, mass spectrometry, are consistent with a two-state equilibrium between the compact (crystal) structure and an extended conformation. (Although ELDOR experiments on the disemiquinone form of CPR have been interpreted in terms of a multistate conformational equilibrium; Hay et al., 2010.) Recent neutron reflectometry studies (Wadsäter et al., 2012) have suggested the coexistence of compact and extended conformations of *Sorghum* CPR incorporated in nanodiscs, suggesting that this equilibrium is also relevant in a membrane environment.

### Domain Movement in the Catalytic Cycle: Synchronization with Electron Transfer

**Figure 5:**
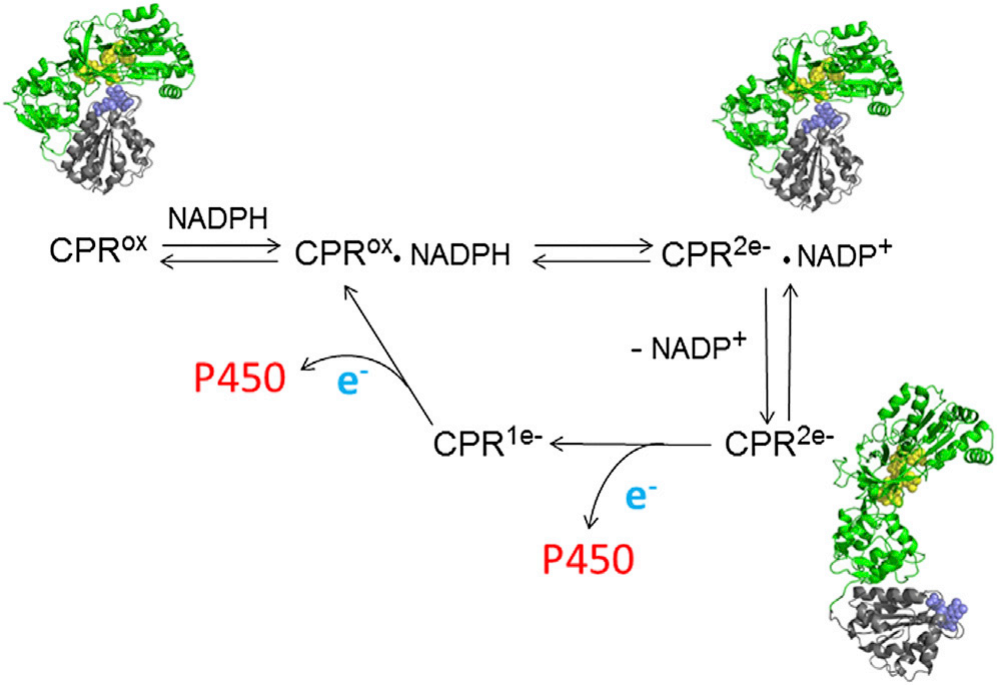
Domain Movement in the Catalytic Cycle of CPR Binding of NADPH to oxidized CPR followed by hydride ion transfer leads to the CPR^2e−^ ⋅ NADP^+^ species, which is predominantly in a compact conformation (Table 1; similar conclusions have been drawn from ELDOR experiments; Hay et al., 2010). This is consistent with the obvious requirement for a compact conformation, with the FAD and FMN in close proximity, for interflavin electron transfer; and, indeed, the rate of interflavin electron transfer increases on binding NADP^+^ (Gutierrez et al., 2002, 2003). Dissociation of NADP^+^ leads to CPR^2e−^, in which the extended conformation is substantially populated (Table 1), facilitating electron transfer to P450 (or cytochrome *c*). The conformations of CPR^ox^ and CPR^2e−^ ⋅ NADP^+^, while both predominantly compact, are likely to be somewhat different. The coenzyme “fragment” 2′5′-ADP, which binds remotely from any interdomain interface, increases the rate of interflavin electron transfer in dithionite-reduced CPR (Gutierrez et al., 2003) and affects the position of the conformational equilibrium (Ellis et al., 2009; Grunau et al., 2006, 2007), suggesting that a tertiary structural change within the FAD domain may occur on binding the 2′5′-ADP moiety of the coenzyme and affect the domain interface. After the transfer of the first electron, the enzyme is likely to return to the compact state transiently to allow transfer of the second electron from FAD to FMN; however, as yet, there is no experimental evidence for the position of the equilibrium in CPR^1e−^. The catalytic cycle shown is based on the assumptions that CPR undergoes a 0-2-1-0 electron transfer cycle and that the four-electron-reduced enzyme is not catalytically relevant (Murataliev et al., 2004).

It is important to note that the SAXS and SANS experiments on different states of CPR allow us to relate the position of the conformational equilibrium to the catalytic cycle of the enzyme, as indicated in Figure 5. Our structural evidence shows clearly that CPR^2e−^ exists to a significant extent in an extended conformation, which would allow P450s and cytochrome *c* to access the FMN in this state of the enzyme. Scrutton and colleagues (Pudney et al., 2011) have also suggested, from fluorescence resonance energy transfer experiments, that reduction of the enzyme leads to formation of an “open” form of the enzyme. These experiments were carried out in phosphate buffer, which binds to the coenzyme site (Grunau et al., 2006; Murataliev et al., 2004), and the existence of multiple sites of labeling and difficulties in deconvoluting the different contributions to the emission response means that they can be interpreted only qualitatively. However, they do provide additional evidence that formation of the two-electron-reduced enzyme is accompanied by domain movement. Binding of coenzyme to CPR^2e−^ shifts the equilibrium back toward the compact form, but judging from the *D*_*max*_ values (Table 1), there is still a larger proportion of the extended form than in the oxidized enzyme. The correlation in the oxidized enzyme between *D*_*max*_ (or the proportion of the extended conformation) and the kinetic parameters for cytochrome *c* reduction, seen both for changes in ionic strength and for the effects of mutations, is consistent with a requirement for the extended conformation for electron transfer to cytochrome *c*. Accurate estimates of the equilibrium constant between the compact and extended conformations are not yet available, but comparison of the *D*_*max*_ value for CPR^2e−^ with those observed at high ionic strength suggests the existence of a finely balanced equilibrium between the compact state, required for intramolecular electron transfer from FAD to FMN, and the extended state, required for intermolecular electron transfer from the FMN to cytochrome *c* or P450.

### A Proposed Mechanism for Domain Movement

**Figure 6:**
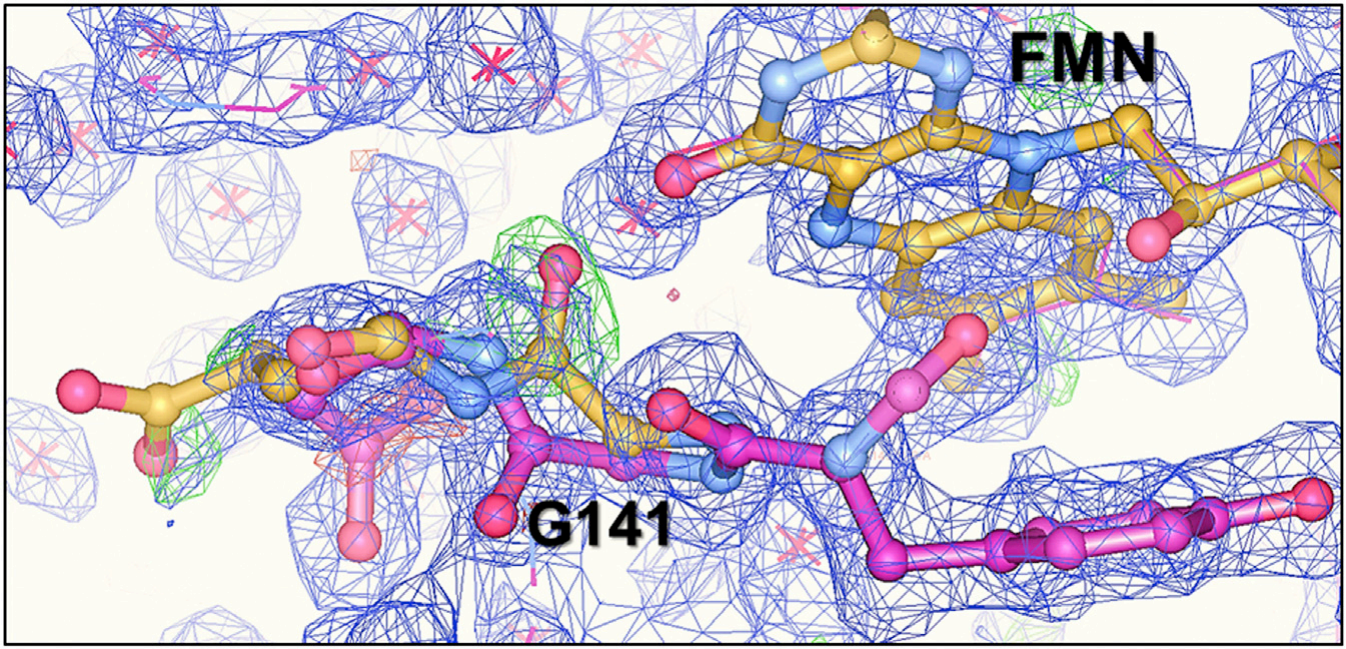
The Structure and Experimental Electron Density of Human CPR in the Vicinity of the FMN Cofactor The structure of human CPR (3QE2) is shown in magenta, and the experimental electron density is shown in blue. The Fo-Fc map is shown in green, contoured at 3σ, and clearly shows a second conformation of the G141 peptide bond, which is as seen in the rat enzyme (1AMO, superimposed in yellow).

In considering possible mechanisms for the redox-linked domain movement in CPR, a natural starting point is the effects of reduction on the flavins (Senda et al., 2009), which are at the domain interface in the compact conformation. Flavin reduction will be accompanied by protonation at N5 of the FMN and, therefore, by altered hydrogen-bonding interactions at this atom. N5 is positioned so that it might form a hydrogen bond to the peptide NH of G141 when the flavin is oxidized and to its carbonyl when the flavin is protonated in the semiquinone and hydroquinone states. A reorientation of this peptide bond would thus be required on formation of the neutral semiquinone and protonation at N5; there is good evidence for this in flavodoxins (e.g., Hoover et al., 1999; Ludwig et al., 1997). In fact, close inspection of the electron density map of the X-ray structure of human CPR (Xia et al., 2011b) supports exactly such a role for G141 in CPR (Figure 6). Density can clearly be seen that shows this peptide bond in two positions, providing evidence for this peptide bond reorientation and raising the possibility of partial reduction in the published crystal structures. The next residue to G141 is E142, which forms part of the interface between the FMN and FAD domains. Thus, changes in the protonation state of N5 on reduction could be transmitted to the interface, as the trigger for domain motion.

In conclusion, the structural evidence reported here provides clear support for the idea that, in CPR, the domain movement necessary for enzyme turnover is not achieved simply by a random diffusive motion of the FMN domain. Rather, domain motion is linked closely to the individual steps of the catalytic cycle of the enzyme, allowing a precise control of electron transfer in this complex system.

## Experimental Procedures

### Materials

NADPH, 2′,5′-ADP, NADP^+^, NADH, dithionite, and horse heart cytochrome *c* were purchased from Sigma-Aldrich. All other chemicals were of analytical grade.

### Protein Expression and Purification

The gene for human fibroblast CPR lacking the N-terminal membrane-anchoring region (a kind gift from Professor C.R. Wolf, University of Dundee) was expressed in *Escherichia coli* BL21 Star and purified as described elsewhere (Ellis et al., 2009). ^2^H,^13^C,^15^N-labeled CPR was produced from cells grown in *E. coli*-OD2 CDN media (Silantes). Protein concentration was calculated using a molar extinction coefficient of ε_450 nm_ = 22,000 M^−1^ cm^−1^. Site-directed mutagenesis was carried out using the Stratagene Quikchange II kit using a standard protocol; custom oligonucleotides were purchased from Eurofins MWG-Operon.

### Enzyme Assay

Cytochrome *c* reduction assays were carried out by following absorbance changes at 550 nm, in 100 mM BES [N,N-bis(2-hydroxyethyl)-2-amino-ethane sulfonic acid], pH 7.0, at 25°C, with 50 μM NADPH and variable cytochrome *c* concentrations. Ionic strength was adjusted by addition of NaCl.

### Solution Scattering Data Collection and Analysis

SAXS measurements were carried out using the SWING beamline at the SOLEIL synchrotron, Saint-Aubin, France. The beam wavelength was set to λ = 1.033 Å. The 17 × 17 cm^2^ low-noise Aviex CCD detector was positioned at a distance of 2,107 mm from the sample, with the direct beam off-centered. The resulting exploitable *q* range was 0.005–0.5 Å^−1^, where *q* = 4π sin θ/λ, and 2θ is the scattering angle. CPR samples at 0.5–10 mg ml^−1^ in 100 mM BES, pH 7.0, were analyzed by direct injection or high-performance liquid chromatography (HPLC) mode. In the first case, they were transferred into the SAXS flowthrough capillary cell, and a series of 50 frames was recorded. In the second case, they were loaded into a size exclusion column (Agilent Bio SEC-3, 300 Å, 4.6 × 300 mm, 3 μm) using an Agilent HPLC system and eluted into the SAXS flow-through capillary cell at a flow rate of 0.2 ml min^−1^. SAXS measurements were collected throughout the whole protein elution time, with a frame duration of 1,000 ms and a dead time between frames of 500 ms. Data processing, analysis, and modeling steps were carried out with PRIMUS (Konarev et al., 2003) and other programs of the ATSAS suite (Konarev et al., 2006). *R*_*g*_ was derived from the Guinier approximation (Guinier, 1939) and, together with the *D*_*max*_, P(*r*), and the forward scattering intensity, was calculated using the indirect Fourier transform method with the program GNOM (Svergun, 1992). Model-independent molecular shapes were calculated using GASBOR (Petoukhov and Svergun, 2003). Twenty independent GASBOR runs were averaged using DAMAVER (Volkov and Svergun, 2003) to obtain a typical molecular shape. Scattering profile simulations from crystal coordinates were carried out using CRYSOL (Svergun et al., 1995).

SANS data were obtained on the LOQ small-angle diffractometer at the ISIS Pulsed Neutron Source (STFC Rutherford Appleton Laboratory, Didcot, UK; http://www.isis.stfc.ac.uk/) (Heenan et al., 1997). This is a fixed-geometry “white beam” time-of-flight instrument that utilizes neutrons with wavelengths between 0.2 and 1 nm. Data are simultaneously recorded on two 2-dimensional position-sensitive neutron detectors to provide a simultaneous *q* range of 0.08−16 nm^−1^. Each sample was placed in a 2 mm path length quartz cuvette and was measured for a total of 1 hr in order to gather data of high statistical precision. Each raw scattering data set was then corrected for the detector efficiencies, sample transmission, and background scattering and converted to scattering cross-section data (δΣ/δΩ versus *q*) using instrument-specific software (Heenan et al., 1989; King and Heenan, 1995). These data were placed on an absolute scale (cm^−1^) using the scattering from a standard sample (a solid blend of hydrogenous and perdeuterated polystyrene) using established procedures (Wignall and Bates, 1987).

### NMR Spectroscopy

Samples of 0.5 mM ^2^H,^13^C,^15^N-labeled CPR were prepared in 30 mM BES, pH 6.5, containing 10% D_2_O. All NMR experiments were performed at 25°C on Bruker AVANCE DRX 600 and AVANCE DRX 800 spectrometers equipped with cryoprobes. Proton chemical shifts were referenced to external 4,4-dimethyl-4-silapentane-1-sulfonic acid; the ^15^N and ^13^C chemical shifts were referenced indirectly using recommended gyromagnetic ratios (Wishart et al., 1995). Spectra were processed with Topspin (Bruker) and analyzed using Collaborative Computing Project for the NMR Community (CCPN) Analysis (Vranken et al., 2005). Three-dimensional HNCO, HNCA, HNCOCA, HN(CA)CO, HNCACB, and HN(CO)CACB experiments were used to assign resonances of CPR, focusing particularly on those of the FMN domain, using standard procedures (Lian and Barsukov, 2011). These partial assignments have been deposited in the Biological Magnetic Resonance Bank under accession number 19301. They are consistent with those of Vincent et al. (2012), as far as can be judged from the spectrum in their Supplementary Material; their assignments are not yet available. It should be noted that their spectra were obtained under different conditions of buffer, pH, and temperature from those used in the present work. All cross-peaks were picked in the CPR spectrum, and the chemical shift difference from the corresponding cross-peak in the spectrum of the FMN domain, Δδ(HN,N), was calculated using the equation

$$\Delta \delta \left(\text{HN,N}\right)=\sqrt{\Delta \delta_{\text{H}}^{2}+\text{0}\text{.}154\Delta \delta_{\text{N}}^{2}},$$

where Δδ = δ_CPR_ – δ_FMN_. The chemical shifts were compared automatically using a computer script without any attempt to adjust the cross-peak mapping between the two spectra. Resonances of a number of residues in the FMN domain of intact CPR (indicated as asterisks in the histogram of Figure 4) could not be identified in any of the triple-resonance spectra, most probably due to exchange broadening.

## Figures and Tables

**Table 1:** Hydrodynamic Parameters for Oxidized and Reduced CPR Obtained from Solution Scattering Experiments

Sample	*R*_*g*_ (SAXS) (Å)	*D*_*max*_ (SAXS) (Å)	*R*_*g*_ (SANS) (Å)
Oxidized	26.4	73	24.8
Dithionite 2e^−^-reduced	28.8	80	29.0
NADPH 2e^−^-reduced	27.6	76	27.8
NADH 2e^−^-reduced	29.3	83	–
Dithionite 2e^−^-reduced + NADP^+^	27.7	78	–

Errors were in the range ±0.1–0.4 Å for SAXS *R*_*g*_ values, ±0.4–0.6 Å for SANS *R*_*g*_ values, and ±1–2 Å for *D*_*max*_ values. See also Figures S1 and S3.

**Table 2:** Kinetic and Hydrodynamic Parameters of CPR Mutants

Protein	K_M_^cytochrome *c*^ (μM)	k_cat_ (s^−1^)	*R*_*g*_ (Å)	*D*_*max*_ (Å)
Wild-type	2.4 ± 0.6	21.2 ± 0.4	26.4 ± 0.1	73 ± 1
K75E/R78E/R108Q	0.46 ± 0.05	44.0 ± 0.5	29.3 ± 0.3	93 ± 2
V233P/E234P	0.7 ± 0.6	26.9 ± 0.3	31.2 ± 0.2	108 ± 2
