# Supplementary material for: Redox-Linked Domain Movements in the Catalytic Cycle of Cytochrome P450 Reductase
Source: Structure. 2013 Sep 3;21(9):1581–9. doi: 10.1016/j.str.2013.06.022 (PMC3763376; doi:10.1016/j.str.2013.06.022)
Supplement: Document S1. Figures S1–S6 and Table S1 [file mmc1.pdf]

Structure, Volume *21*

Supplemental Information

Redox-Linked Domain Movements

in the Catalytic Cycle

of Cytochrome P450 Reductase

Wei-Cheng Huang, Jacqueline Ellis, Peter C.E. Moody, Emma L. Raven, and Gordon C.K. Roberts

Inventory of Supplemental Information

Figure S1
Small angle X-ray scattering of CPR in the oxidised and reduced states; related to Figure 1 and Table 1.

Figure S2

Effects of X-ray exposure on the optical spectra of CPR; related to Figure 1 and Table 1.

Figure S3

Guinier plots of SANS data for oxidised and 2-electron-reduced CPR; related to Table 1.

Figure S4

Differences in backbone chemical shifts of residues in the FMN domain between intact CPR and the isolated domain; related to Figure 2.

Figure S5

Sequence alignment of residues in helices B, F and I of CPR; related to Figure 3.

Figure S6

Effects of increasing ionic strength on the kinetic parameters for cytochrome *c* reduction by CPR; related to Figure 4.

Table S1
Effects of ionic strength on the hydrodynamic parameters of oxidised CPR; related to Figure 4.

Supplemental Information

Figure S1

Small angle X-ray scattering of CPR in the oxidised and reduced states; related to Figure 1 and Table 1.

Scattering curves are displaced vertically for clarity. Blue, oxidized enzyme; red, enzyme reduced to the two-electron level by careful anaerobic titration with dithionite, monitored by optical absorption spectroscopy; yellow, enzyme reduced to the two-electron level by addition of stoichiometric NADPH. Error bars (SD) are included for each data point.

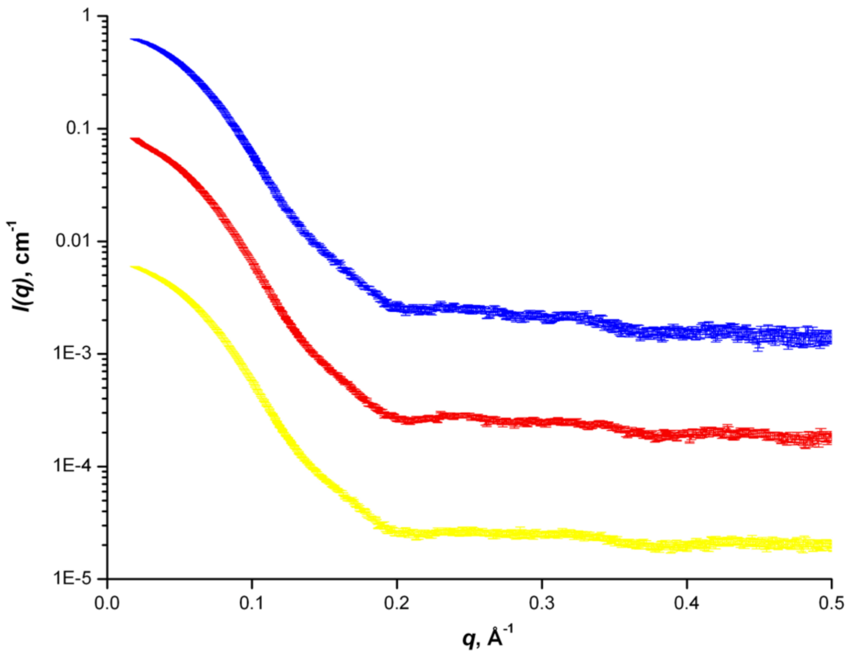

Figure S2

Effects of X-ray exposure on the optical spectra of CPR; related to Figure 1.

Blue, spectrum of oxidised CPR in a capillary in an X-ray generator before exposure to X-rays; *red*, spectrum after exposure to X-rays (λ: 1.5418 Å, flux: 3.2×10^{8} photons/cm^{2}/sec for 16 hours). *Green*, control spectrum of oxidised CPR, scaled to the spectrum in the capillary. The increase in absorbance at ~590nm characteristic of semiquinone formation is clearly visible following X-ray exposure.

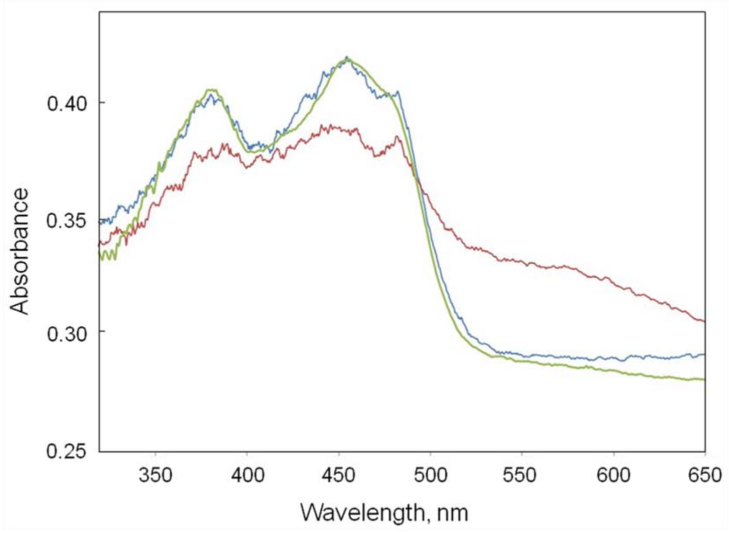

Figure S3

Guinier plots of SANS data for oxidised and 2-electron-reduced CPR; related to Table 1.

x, oxidized CPR; **+**, dithionite two-electron-reduced CPR; **●**, NADPH two-electron-reduced CPR. Error bars (SD) are, in many cases, smaller than the size of the data points.

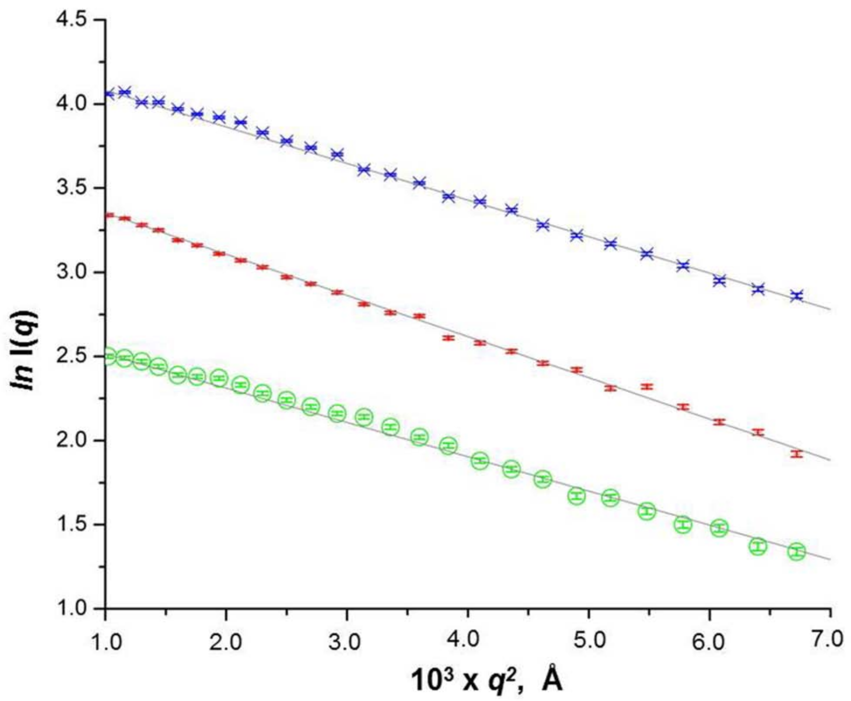

Figure S4

Differences in backbone chemical shifts of residues in the FMN domain between intact CPR and the isolated domain; related to Figure 2.

Combined ^{1}H, ^{15}N shift differences ($\Delta\delta(\mathrm{HN,N}) = \sqrt{\Delta\delta_{\mathrm{H}}^2 + 0.154\Delta\delta_{\mathrm{N}}^2}$) are shown as a function of sequence position. Residues whose cross-peaks were unobservable due to line-broadening are indicated by asterisks, and proline residues are indicated by P.

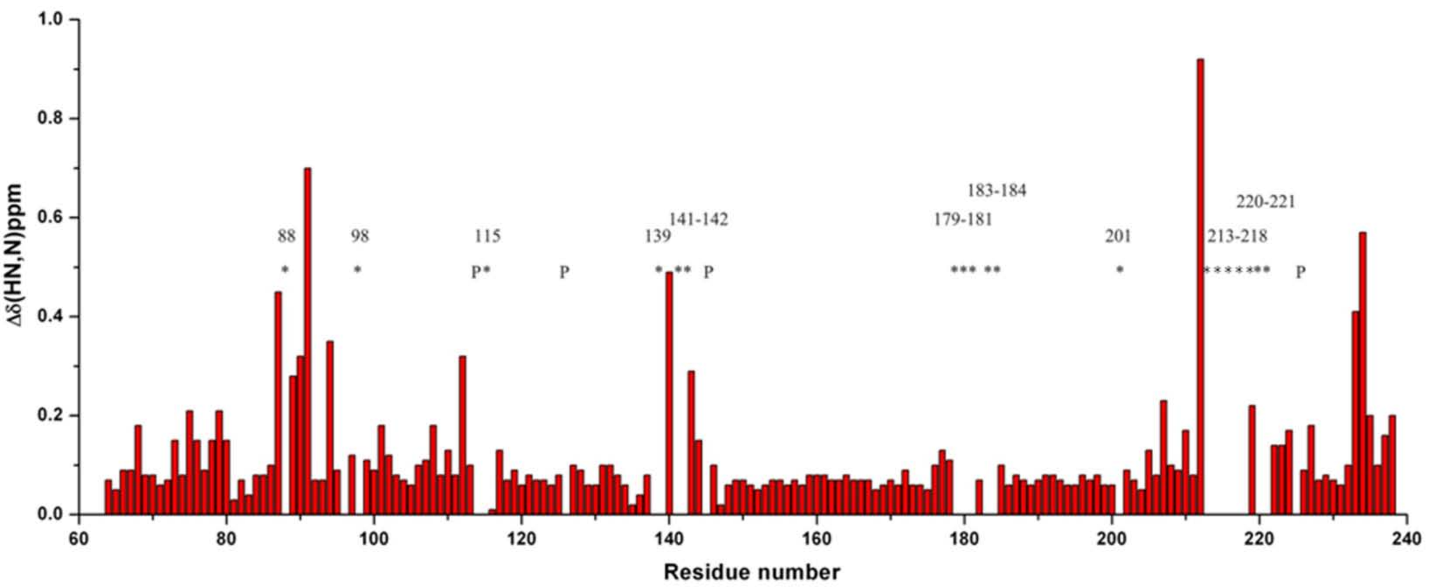

Figure S5

Sequence alignment of residues in helices B, F and I of CPR; related to Figure 3.

The alignments were carried out using the DbClustal server (http://bips.u-strasbg.fr/PipeAlign/ jump_to.cgi?DbClustal+noid). Residue numbers are those for the human enzyme used in the present paper. Conserved residues making polar inter-domain contacts in the *compact* conformation (represented by the crystal structure) are highlighted in cyan, with functionally similar residues shaded grey. Conserved residues making polar inter-domain contacts in the model of the *extended* conformation are highlighted in green, with functionally similar residues shaded grey. E213, highlighted yellow, makes inter-domain contacts in both conformations, with T383 in the compact conformation and with Y387 in the extended conformation.

	Helix B	Helix F	Helix I
	90 100	212 231	382 392
Human	GSQTGTAEEFANRLSKDAHR.YGMR	DGNLEEDFITWREQFWPAVCEHFG	PPRTNVLYELAQYASEP
Chimpanzee	GSQTGTAEEFANRLSKDAHR.YGMR	DGNLEEDFITWREQFWPAVCEHFG	PPRTNVLYELAQYASEP
Macaca	GSQTGTAEEFANRLSKDAHR.YGMR	DGNLEEDFITWREQFWPAVCEHFG	PPRTNVLYELAQYASEP
Rat	GSQTGTAEEFANRLSKDAHR.YGMR	DGNLEEDFITWREQFWPAVCEFFG	PPRTNVLYELAQYASEP
Mouse	GSQTGTAEEFANRLSKDAHR.YGMR	DGNLEEDFITWREQFWPAVCEFFG	PPRTNVLYELAQYASEP
Dog	GSQTGTAEEFANRLSKDAHR.YGMR	DGNLEEDFITWREQFWPAVCEHFG	PPRTNVLYELAQYASEP
Horse	GSQTGTAEEFANRLSKDAHR.YGMR	DGNLEEDFITWREQFWPAVCEHFG	PPRTNVLYELAQYASEP
Cow	GSQTGTAEEFANRLSKDAHR.YGMR	DGNLEEDFITWREQFWPAVCEHFG	PPRTNVLYELAQYASEP
Pig	GSQTGTAEEFANRLSKDAHR.YGMR	DGNLEEDFITWREQFWPAVCEHFG	PPRTNVLYELAQYASEP
Rabbit	GSQTGTAEEFANRLSKDAHR.YGMR	DANLEEDFITWREQFWPAVCEHFG	PPRTNVLYELAQYAADP
Chicken	GSQTGTAEEFANRLSKDAHR.YGLR	DGNLEEDFITWREQFWPAVCEHFG	PPRTNVLHELAQYATDT
Xenopus laevis	GSQTGTGEEFANRLAKDAHR.YGVR	DGNLEEDFITWREQFWPAVCEHFG	PPRTNVLYELAQYATDS
Danio rerio	GSQTGTAEEFSNRLAKDAHR.YGMK	DGNLEEDFVSWREQFWPAVCEHFG	SPRTNVLYELAQYASDP
D. melanogaster	GSQTGTGEEFAGRLAKEGIR.YRLK	DANIEDDFITWKDRFWPAVCDHFG	IPRTHILKELAEYCTDE
Musca domestica	GSQTGTAEEFAGRLAKEGLR.YRMK	DANIEDDFITWKDRFWPSVCDFFG	IPRTHILKELAEYCSDE
C. elegans	GSQTGTAEEMSGRLAKDLTR.YTKK	DANLEEDFMIWREAFLPKVAEEFG	PVKSHVLKAISEYCTDD
D. discoides	GTQTRTAEDFSRIIEKECKK.IGIP	DATLEEDFNRWKKDMWPVVCKFLG	PVRKSVLRALAESTTNE
S. pombe	GSQTGTAEDYAHRLAKDITASFGVN	EGMLEEDYLEWKEDFLADFAGEFE	VVSRQFLSAIAVYAPTA
S. cerevisiae	ASQTGTAEDYAKKFSKELVAKFNLN	AGTTDEDYMAWKDSILEVLKDELH	PVSRQLFSSLIQFAPNA

Figure S6

Effects of increasing ionic strength on the kinetic parameters for cytochrome *c* reduction by CPR; related to Figure 4.

Where error bars (standard deviations) are not visible, they are smaller than the size of the data point. The quantitative differences from earlier results (Jang et al., 2010; Sem and Kasper, 1995) can be attributed to differences in buffer and pH and, particularly, to the use in the earlier work of an affinity column for purification of the enzyme, resulting in a significant amount of 2′-AMP, used for elution from the column, remaining bound to the enzyme (Grunau et al., 2006).

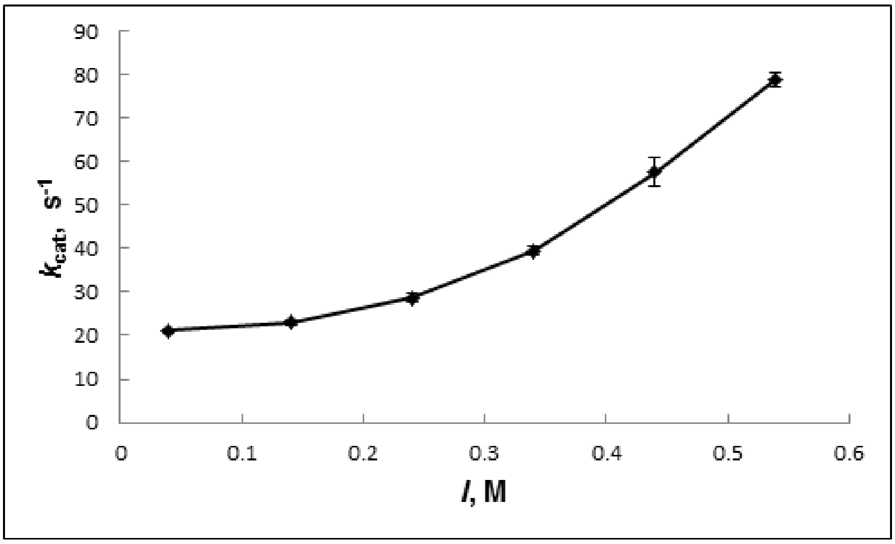

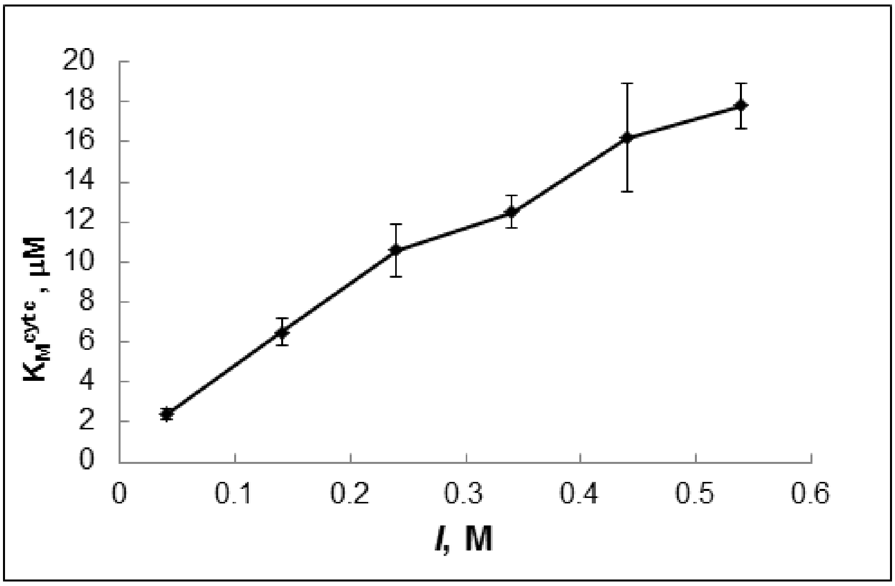

Table S1

Effects of ionic strength on the hydrodynamic parameters of oxidised CPR; related to Figure 4.

Ionic strength (M)	R_g (Å)[a]	D_{max} (Å)[a]
0.04	26.4	74
0.14	26.9	75
0.24	27.1	75
0.34	28.3	84
0.44	29.4	99
0.54	32.5	108

[a] Errors were in the range ± 0.1-0.4Å for R_g values and ± 1-2Å for D_{max} values.
